# Dynamic–Mechanical and Decomposition Properties of Flax/Basalt Hybrid Laminates Based on an Epoxidized Linseed Oil Polymer

**DOI:** 10.3390/polym13040479

**Published:** 2021-02-03

**Authors:** Dana Luca Motoc, Jose Miguel Ferri, Santiago Ferrandiz-Bou, Daniel Garcia-Garcia, Rafael Balart

**Affiliations:** 1 Department of Automotive and Transport Engineering, Transilvania University of Brasov (UniTBv), 1 Politehnicii Street, 500024 Brasov, Romania; 2 Institute of Materials Technology (ITM), Universitat Politècnica de València (UPV), Plaza Ferrandiz y Carbonell s/n, 03801 Alcoy, Alicante, Spain; joferaz@epsa.upv.es (J.M.F.); sferrand@mcm.upv.es (S.F.-B.); dagarga4@epsa.upv.es (D.G.-G.); rbalart@mcm.upv.es (R.B.)

**Keywords:** epoxidized linseed oil (ELO), flax, basalt, dynamic–mechanical, green composites, mass loss, resin transfer molding (RTM)

## Abstract

This contribution focuses on the development of flax and flax/basalt hybrid reinforced composites based on epoxidized linseed oil (ELO) resin, exploiting the feasibility of different ratios of glutaric anhydride (GA) to maleinized linseed oil (MLO) in the hardener system (50:0, 40:10 and 30:20 wt.%) to provide crosslinked thermosets with balanced properties. The hybrid laminates have been manufactured by resin transfer molding (RTM) and subjected to dynamic–mechanical (DMA) and thermal gravimetry (TGA) analysis. The presence of glutaric anhydride (GA) resulted in hard and relatively brittle flax and flax/basalt laminates, whose loss moduli decreased as the number of basalt plies diminished. Furthermore, the increase in MLO content in the GA:MLO hardener system shifted the glass transition temperatures (*T*_g_) from 70 °C to 59 and 56 °C, which is representative of a decrease in brittleness of the crosslinked resin. All samples exhibited two stages of their decomposition process irrespective of the MLO content. The latter influenced the residual mass content that increased with the increase of the MLO wt.% from 10 to 30 wt.%, with shifts of the final degradation temperatures from 410 °C to 425 °C and 445 °C, respectively.

## 1. Introduction

Development of niche products to mitigate global warming effects and implement viable solutions for harmful emission reduction is an ongoing concern across a wide range of industry players, government agendas and policies, environmental protection agencies’ initiatives and researchers’ studies [1,2,3,4]. Material substitution was sought as a solution to emission reduction, lightweighting or depletion of fossil resources and was successfully used to address issues regarding sustainability, environmentally friendly products, biodegradability and availability [5,6]. Recent research trends envisage the replacement of both conventional synthetic reinforcements and resins in polymer-based composites with plant fibers and bio-based or green polymer resins [7,8]. Numerous studies report on naturally reinforced, thermosetting and thermoplastic composites made out of plant-based fibers like flax, hemp, kenaf, sisal, jute and ramie, among others [9,10,11,12,13]. These studies cover a wide range of topics from manufacturing techniques (e.g., manual lay-up and resin transfer molding) to material properties (e.g., mechanical, dynamical, electrical, thermo-physical and flammability), natural-fibers surface conditioning, fiber–matrix interface issues and prospective applications (e.g., automotive, construction and building, marine, aerospace and food industry) such as coatings, adhesives, inks, resins and composites.

An increasing interest in bio-based resin development can be traced from the scientific contributions on thermosetting polymers for natural reinforcement composites. These were synthetized as bio-based epoxy resins by addition of vegetable oils (e.g., soybean, linseed, canola, karanja and vernonia), furans, tannins, cardanol or natural rubber. The hardeners deployed for crosslinking processes covered a wide selection of bio-based epoxy curing agents, including amines and amine derivatives, anhydrides, carboxylic acids, phenols, polyphenols, etc. [14,15,16,17,18,19,20]. Vegetable oils (VOs) are a promising source for bio-based materials and additives for the polymers industry. VOs consist of three fatty acids, chemically attached to a glycerol backbone leading to a typical triglyceride structure. The main feature of VO is unsaturation, which can be selectively converted into groups (e.g., hydroxyl, oxirane, maleic anhydride and acrylate) to tailor the desired reactivity. These chemically modified VOs find increasing applications in the polymer industry such as their use in plasticizers, chain extenders, compatibilizers, coatings and thermosetting resins [21,22,23,24,25,26]. Epoxidation is one of the most usual chemical modifications of VO, which leads to epoxidized vegetable oils (EVOs) with growing uses in plasticization and thermosetting resins [27,28,29,30,31]. Currently, it is possible to find several EVOs at commercial scale as epoxidized soybean oil (ESBO) and epoxidized linseed oil (ELO), with main applications in the plasticization industry. Epoxidized linseed oil (ELO) is extensively used in thermoset applications as they not only are cost effective and ecofriendly but also have a low level of toxicity [3,32]. Major disadvantages of commercially available ELOs include their high viscosity (1000–2000 cP at 10 °C) and lack of carbon–carbon double bonds if fully epoxidized along with the transformation into homogeneous clear liquids above room temperature (>30 °C). On the other hand, partially ELOs are homogeneous liquids with low viscosities and melting points below room temperature. Their epoxy functionality varies depending on the average number of carbon–carbon double bonds per triglyceride molecule in the nonfunctionalized linseed oil and their degree of epoxidation [16,33,34]. As oxirane groups in ELO are not located in terminal positions, the crosslinking process usually needs moderate-to-high temperatures and anhydride-based hardener systems.

Common curing agents deployed for bio-based resin crosslinking include amines and amine derivatives (e.g., polyamines, aromatic, furanic and amino acids), anhydrides, phenols and polyphenols. Anhydrides were among the preferences against amines considering their higher glass transition temperatures, low viscosity, very low exothermic reactions and long pot life [17]. For example, cycloaliphatic monoanhydride, phthalic anhydride (PA), pyromellitic dianhydride (PMDA), methyltetrahydrophthalic anhydride (MTHPA), methylhexahydrophthalic anhydride (MHHPA) or methyl nadic anhydride (MNA) have been delivered as novel hardener formulations [16,32,35]. The last three cyclic anhydrides are liquids at room temperature, and this has a positive effect on handling the mixtures before the curing process. Partially bio-based epoxy resins with low temperature hardening systems can be found at commercial scale. Usually, the hardener systems for a low temperature profile curing are based on amines and are increasingly used as matrices in polymer composites [36].

The research groups of Altuna et al. (2011) and Kumar et al. (2017) reported on thermal and mechanical properties of epoxidized soybean oil (ESO) crosslinked with MTHPA in the presence of 1-methyl imidazole (1-MI) and MHHPA in the presence of 2-methyl imidazole (2-MI), respectively [18,37]. Their results reveal similar values of the loss moduli accounting for a 20% ESO in the epoxy resin as retrieved at 30 °C, 2.41 and 2.18 GPa, respectively. On the other hand, the glass transition temperatures reported (*T*_g_) were highly different: 102 and 143 °C, respectively. Both groups concluded the effect of ESO content on the *T*_g_ values, as the content increase lowers the *T*_g_ values.

In this investigation, the papers of Samper et al. (2015) and Fombuena et al. (2019) were referred to since they explored the mechanical and thermal properties of ELO bio-based resin crosslinked with MNA or MNA and maleinized linseed oil (MLO) as a new formula hardener system, respectively [38,39]. In addition, identical process parameters have been deployed as already optimized. From their findings, it was shown that flax-fiber reinforcements revealed improved mechanical properties in the absence of any surface treatments, having the presence of MLO in the hardener system. Next, the MLO content highly decreased the *T*_g_ values, in the range of 50 °C, as retrieved from the loss tangent curves in dynamic mechanic analysis performed in shear mode.

Additionally, Samper et al. (2015) reported on the mechanical properties of basalt-reinforced ELO-based composite crosslinked with MNA in the presence of 1-methylimidazole (1-MI) [39]. Their results revealed increased mechanical properties (i.e., about 10% to 30%), both in tensile and flexure, on composites having their reinforcements previously conditioned with silane derivatives, amino and glycidyl, compared with thermally treated ones.

This contribution reports on the manufacturing of flax and flax/basalt fibers reinforced ELO composites by resin transfer molding, deploying a new environmentally friendly anhydride system as a hardener. Thus, glutaric anhydride (GA) in combination with maleinized linseed oil (MLO) has been considered to develop novel formulations to crosslink the ELO bio-based resin. The effect of the crosslinker on the dynamic–mechanical and thermal degradation properties of the resulting composites has been investigated and debated.

## 2. Materials and Methods

### 2.1. Materials Selection

**Scheme 1:**
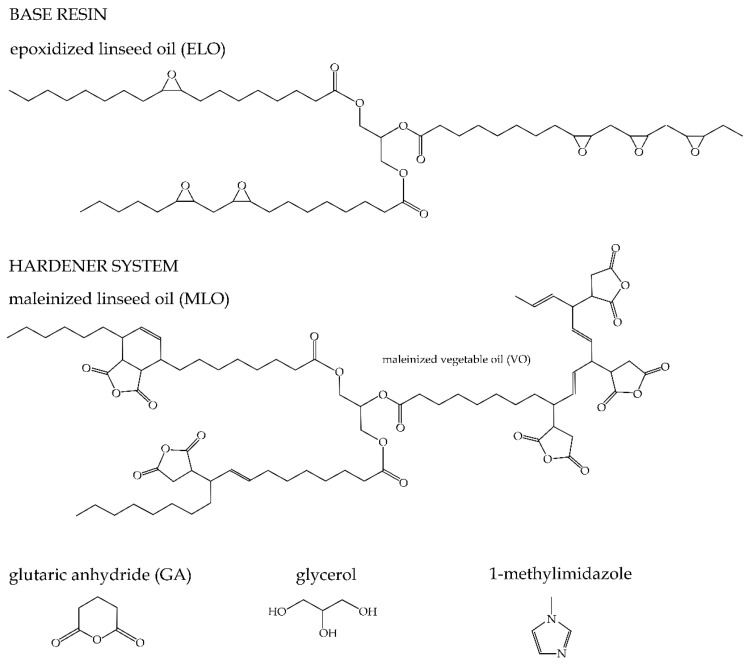
Plot representation of the chemical structure of all components in the epoxidized linseed oil (ELO)-based thermosetting resins as base materials for composites manufacturing.

An epoxidized linseed oil (ELO, 99.5%) supplied by Traquisa S.A (Barcelona, Spain) was selected as the polymer matrix to deliver the final laminates. This ELO is characterized by an epoxy equivalent weight (EEW) of 178 g equiv^−1^. The bio-based epoxy resin was crosslinked with a mixture of glutaric anhydride (GA) and maleinized linseed oil (MLO) under a ratio of 50:50 wt.% between the resin and hardener system. The GA (G3806, 95%) was supplied by Sigma Aldrich (Madrid, Spain) having an anhydride equivalent weight (AEW) of 114.10 g equiv^−1^ while the MLO was acquired from Vandeputte Group (Deerlijk, Belgium) posing an acid value within 105–130 mg KOH g^−1^ and an average anhydride equivalent (AEW) of 203 g equiv^−1^. The base ELO resin was mixed with the hardener system (MLO/GA) under a constant ratio of 50:50 wt.% to cover different molar ratios since MLO provides flexible crosslinked polymers while GA leads to rigid/brittle crosslinked structures. Several mixing procedures by wt.% were formulated prior to delivery of the final hardener blend (e.g., 50:0, 40:10 and 30:20 wt.% which correspond to EEW:AEW ratios of 0.87, 1.01 and 1.15, respectively). After homogenization of the ELO/hardener system, additional 1 wt.% glycerol and 2 wt.% 1-methylimidazole, both supplied by Sigma Aldrich (Madrid, Spain), were added, stirred until homogenization and, subsequently, subjected to the curing thermal cycle. The chemical structure of all components is shown in Scheme 1.

The reinforcements selected for study were flax and basalt fibers. Flax fabric (F) was supplied by Hilaturas Ferre S.A. (Alicante, Spain) as coupons with a surface density of 175 g m^−2^, whereas the basalt fabric (B) was delivered by Basaltex (Marureel, Belgium), with an area weight of 220 g m^−2^. Plain weave pattern was chosen for both fabrics.

### 2.2. Composite Manufacturing

A conventional resin transfer molding (RTM) technique was deployed for the ELO blend-based laminates (dimensions: 300 mm × 300 mm) manufacturing by aid of a Hypaject MKII from Plastech Thermoset Tectonics Ltd. (Gunnislake, UK). Process conditions were similar to those reported by Samper et al. and Fombuena et al. [38,39].

Different composite architectures were delivered by the various stacking of four layers of constitutive, both natural and synthetic. Thus, the following codes were considered for the stacking sequences and further used for sample identification: FFFF, FBBF, BFFB and BBBB, respectively.

The overall fiber loading fluctuated within 55.3 ± 0.5 wt.% for all architectures while the sample thickness fluctuated around 3 ± 0.15 mm. Good-quality surfaces were obtained on all laminates with a roughness appearance on those having the flax fabrics layered as the outermost surfaces.

### 2.3. Composite Characterization

Viscoelastic properties of ELO-based specimens were retrieved in accordance with the ASTM D5023:2007 [40] standard by a dynamic mechanical analyzer DMA 242 C from Netzsch GmbH (Selb, Germany). Samples were subjected to 3-point bending deformation mode at an oscillating frequency of 1 Hz from −50 to 200 °C. Composite specimens 50 × 10 × 3 mm^3^ (L × l × h) were scanned in the dynamic temperature ramp mode at a heating rate of 3 K min^−1^ to avoid all artifacts. The Proteus^®^ software was used to operate the instrument and analyze the experimental data.

Thermal decomposition was studied by thermogravimetric analysis. Measurements were performed using an STA 449 F3 Jupiter^®^ device from Netzsch GmbH (Selb, Germany) at a heating rate of 10 K∙min^−1^, under a nonoxidative atmosphere (N_2_) at a 20 mL∙min^−1^ flow rate, in accordance with the ISO 11358-1:2014 standard [41]. The microbalance had a precision of ±0.1 µg. Alumina crucible (70 µL) was used to hold individual composite excerpt (approx. 15–20 mg) that was further subjected to heating within a 25–700 °C temperature range.

## 3. Results and Discussion

**Figure 1:**
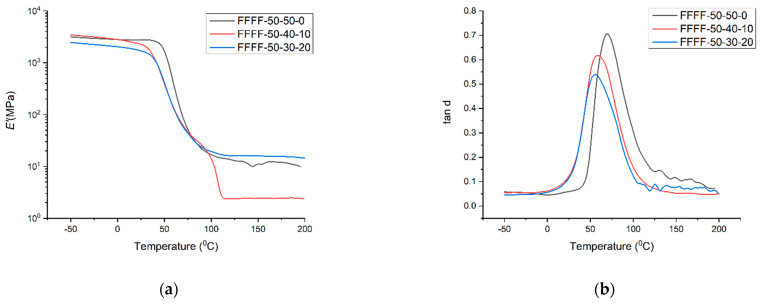
Dynamic–mechanical properties of flax-fiber-reinforced (FFFF) composites based on different epoxidized linseed oil:glutaric anhydride:maleinized linseed oil (ELO:GA:MLO) blends: (**a**) storage modulus evolution with temperature and (**b**) loss tangent evolution with temperature.

Prior to debating the dynamic mechanical properties of composite samples based on ELO epoxy resin, an insight into the influence of MLO co-hardener content upon the property under consideration is given. Glutaric anhydride is a very promising cyclic anhydride from several standpoints. On one hand, it can be bio-derived through its corresponding dicarboxylic acid and, on the other hand, its melting temperature is relatively low, in the 53–57 °C range [42]. Nevertheless, epoxy resins crosslinked with GA are brittle [43]. To overcome this, the present work suggests the use of a flexible anhydride, namely maleinized linseed oil (MLO), in the hardener system. Figure 1a,b shows the evolution of both storage modulus (*E*′) and damping factor or loss tangent (tan δ) with the temperature increase for several flax specimens based on ELO epoxy resin accounting for a different GA:MLO ratio. As can be seen, two transitions (i.e., glass and leathery to rubbery) and three different states (i.e., glassy, leathery and rubbery) can be identified for all combinations. Further, a higher MLO wt.% content in the hardener mixture lowers the specimen’s rigidity under dynamic loading conditions. Thus, addition of MLO in 10 wt.% amounts results in 17.7% and 28.4% decreases, respectively, of flax-reinforced composite elastic moduli retrieved at 25 °C (i.e., temperature of use). The glass transition region is relatively narrow, covering the 50–75 °C temperature range. In addition, both magnitudes decrease and shift toward lower temperature values encountered in the damping factor peaks (see Figure 1b). Thus, addition of 10% and 20% of MLO as a co-hardener results in a negative shift of *T*_g_ values to 59 and 56 °C, respectively, which represents a 15.7% and 20% decrease with respect to the reference (ELO:GA) based composite. Additionally, the addition of MLO results in the loss tangent curve slightly broadening. All curves have a symmetric gaussian shape of the tan δ peak that is an indication of the homogeneous distribution of relaxation times in polymer motion [16]. Table 1 lists the glass transition temperatures (*T*_g_) and peak intensities retrieved from tan δ curves, along with the crosslinking densities and storage modulus values retrieved at 25 °C. The crosslinking density (ν_e_) of the laminates was estimated according to the rubbery elastic theory and considering the storage modulus (*E*′) in the rubbery plateau region (*T*_g_ + 40 °C), gas constant (R, 8.314 J∙(K∙mol)^−1^) and absolute temperatures (K) [44,45].

**Figure 2:**
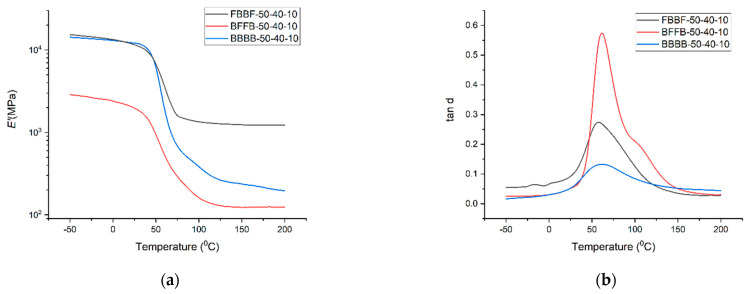
Dynamic–mechanical properties comparison on different stacked sequences of flax (F) and basalt fibers (B) based on 10 wt.% MLO co-hardener: (**a**) storage modulus evolution with temperature and (**b**) loss tangent evolution with temperature.

Further development focused on mechanical properties of different stacked sequences of flax and basalt fibers under dynamic loadings accounting for a 10 wt.% of MLO in the ELO crosslinking process. The ELO:GA:MLO blend under a 50:40:10 ratio is seen to exhibit the best compromise on elastic and viscous components as described earlier and selected consequently. Figure 2a,b shows the evolution with the temperature increase of the storage modulus and damping curves of the composite architectures developed based on the combination above.

As can be seen, the DMA curves follow the general trend of crosslinked polymers. This can be seen especially with the rubbery plateau of the corresponding plots (see Figure 2a). Glass transition temperatures (*T*_g_) identified with loss tangent peak (n. single peak maximum ascribed to the α-relaxation) for flax- and flax/basalt-reinforced composites herein exhibit relatively small discrepancies, fluctuating in the vicinity of 60 °C. The latter can be regarded with the presence of the MLO in the resin blend with a stabilizing effect upon the specimens’ viscoelastic behavior as reported by Fombuena et al. [38]. Further, laminates based solely on flax-fabric reinforcement pose the lowest values of both elastic modulus and tan δ peak intensities. This result confirms a decreased damping ability of flax composite in the vicinity of *T*_g_ compared with its hybrid counterparts. Moreover, the addition of MLO in 10 and 20 wt.% as a co-hardener to the ELO resin blend results in a negative shift of *T*_g_ to 59 and 56 °C, respectively, as reported above. The calculated ν_e_ values increase with the addition of MLO wt.% with the resin blend, which indicates a considerably increased crosslinking density.

A closer view on the storage modulus evolution with the glassy plateau reveals discrepancies between FBBF, BFFB and BBBB architectures. Therefore, the storage modulus of the BFFB architecture doubles compared with its counterpart. This can be assigned to the fiber–matrix interaction that is poor in the case of lignocellulosic flax-fabric-reinforced ELO blend in the presence of the MLO co-hardener.

**Figure 3:**
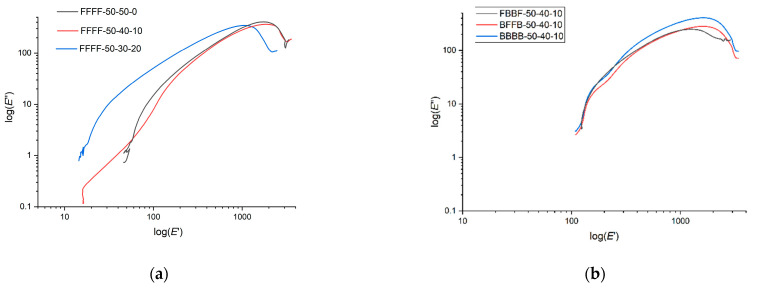
Cole–Cole plots: (**a**) flax-fiber-reinforced composites based on different ELO:GA:MLO blends and (**b**) flax- and flax/basalt-reinforced composites based on 10 wt.% MLO co-hardener.

The heterogeneity of the hybrid specimens herein was investigated using the Cole–Cole diagrams. These are used as indicators for the homogeneity of the bio-based resin systems. In Figure 3a,b, it can be seen that the Cole–Cole plots of the loss modulus (log *E*″) as a function of the storage modulus (log *E*′) for the flax-fiber-reinforced composites based on different ELO:GA:MLO blends along with that of hybrid composite structures account for a 10 wt.% of MLO in the ELO resin. Departure from the ideal semicircular shape can be seen in all composite architectures regardless of the resin system, especially in flax-fiber-reinforced architectures.

At a glance, 10 wt.% MLO added as a co-hardener in the ELO resin does not significantly change the overall behavior of flax-reinforced composites in shock and vibrations. Supplementing the MLO wt.% in the stoichiometric composition of the hardener changes the shape of the Cole–Cole diagram, as seen in Figure 3a. Therefore, increasing the MLO content in the hardener combination leads to a curve shape closer to the ideal semicircular shape with direct consequences on both laminates’ overall stiffness and dynamic behavior. For hybrid flax/basalt laminates based on the same MLO wt.% content, there are no significant differences on their Cole–Cole curve shapes as seen in Figure 3b. On the other hand, flax-reinforced composites exhibit a departure that decreases the amount of energy that can be dissipated throughout the structure under dynamical loading conditions.

**Figure 4:**
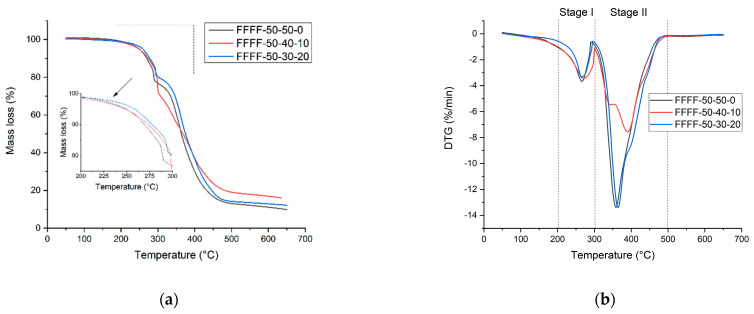
Thermal degradation profiles of flax-fiber-reinforced composites based on different ELO:GA:MLO blends: (**a**) weight loss (TG) and (**b**) rate of weight loss (DTG).

**Figure 5:**
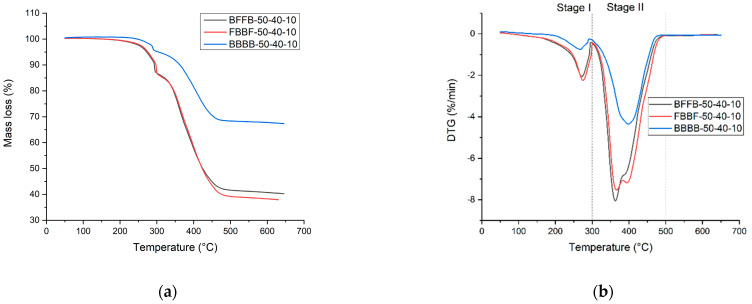
Thermal degradation profiles of flax- and flax/basalt-reinforced composites based on 10 wt.% MLO co-hardener: (**a**) weight loss (TG) and (**b**) rate of weight loss (DTG).

The thermal degradation profiles as determined by thermogravimetric analysis (TGA) of the flax- and flax/basalt-reinforced bio-based thermoset ELO resin crosslinked with different compositions of GA and MLO as a co-hardener are shown in Figure 4 and Figure 5a,b and summarized in Table 2 and Table 3.

The ELO-based flax laminates, with/without MLO added as co-hardener, reveal a two-stage thermal degradation process occurring within 200–300 °C and 300–500 °C ranges, respectively, leaving between 10% and 15% char residues behind as measured at 650 °C. Similarities of the TG and DTG curves in Figure 4 indicate that the thermal decomposition process is dominated by flax fibers with small discrepancies due to the presence of GA and MLO co-hardener. Degradation curves are typical to natural reinforcements, as shown by decomposition temperatures at peaks of the DTA curves. The first peak in the range of 265–270 °C corresponds to the decomposition of hemicellulose (mass loss about 20%) while the second peak (around 360 °C, mass loss about 45%) is due to decomposition of cellulose [10,12]. Prior hemicellulose decomposition, waxes, pectin and other water-soluble substances are gradually removed. Char residues are due to the lignin content of flax-fiber structure [46]. Degradation of ELO blend resin occurs overlapped with the flax-fiber degradation, undergoing high mass loss up to 300 °C that can be explained by spontaneous fatty acid chain scission resulted from the reaction of epoxide rings with the anhydride hardener [47].

Presence of basalt reinforcement in the hybrid architecture does not change the TG and DTG curve profiles, but the char residues are left in the device crucible in relatively high amounts (40–45%). In addition, differences between first peak temperature values from flax to flax/basalt hybrid architectures are about ±5 °C that may reflect acceptable measurement errors and thus are nonquantifiable in the thermal stability behavior of composites herein.

## 4. Conclusions

A new environmentally friendly anhydride system, composed of glutaric anhydride (GA) and maleinized linseed oil (MLO) into various wt.% ratio combinations, was proposed for crosslinking a bio-based epoxidized linseed oil (ELO) resin. Further, various stacked sequences of flax (F) and basalt (B) reinforcements, manufactured by resin transfer molding (RTM), were delivered as 4-ply hybrid architectures (FBBF, BFFB) based on the novel formula system selected (ELO:GA:MLO as 50:40:10 wt.%). Insights into dynamic mechanical and thermal degradation properties of these bio-based thermosetting hybrid composites revealed differences due to stacking sequences compared with their counterparts (FFFF or BBBB). Thus, while the loss tangent peak intensity, crosslinking density and storage modulus are increasingly higher with basalt fabrics layered outermost, there are no significant differences on the Cole–Cole distribution, and all composites withstand shock and vibrations comparably. On the other hand, thermal degradation seems to be unaffected by constitutive stacking sequence, revealing no significant differences on degradation temperature’s values (e.g., initial, highest and final), as well as on the mass change and residual mass.

To summarize, the green composites herein, developed using a new synthesis route for their thermoset ELO resin, appear to be attractive for practical applications accounting for both relatively low manufacturing costs and enhanced investigated properties.

## Figures and Tables

**Table 1:** Glass transition temperature and storage modulus of epoxidized linseed oil:glutaric anhydride:maleinized linseed oil (ELO:GA:MLO)-based composites.

Sample Architecture	Resin Blend (ELO:GA:MLO)	*T*_g_ (°C)	Peak Intensity of tan δ Curve	Crosslinking Density (×10^3^ mol m^−3^) ^1^	Storage Modulus (GPa) ^2^
FFFF	50:50:0	70	0.705	1.614	2.757
	50:40:10	59	0.617	2.120	2.268
	50:30:20	56	0.538	2.054	1.954
FBBF	50:40:10	59	0.274	15.369	2.873
BFFB		61	0.573	40.412	11.125
BBBB		62	0.132	142.636	11.575

^1^$\nu_{e}=E\prime /3RT$; ^2^ retrieved at 25 °C; F—flax layer; B—basalt layer.

**Table 2:** Thermal degradation results of flax and flax/basalt composites based on ELO:GA:MLO.

Mass Loss Data	FFFF			FBBF	BFFB	BBBB
	50:50:0	50:40:10	50:30:20	50:40:10		
*T*_onset_ (°C)	300.9	304.6	305.3	303.6	302.3	324.6
*T*_mid_ (°C)	354.3	365.3	339	371.9	366.0	384.8
*T*_max1_ (°C)	266.1	269.8	268.5	270.6	272.0	267.5
*T*_max2_ (°C)	362.4	367.6	383.2	359.2	364.1	398.2
*T*_end_ (°C)	411.2	423.9	445.8	434.8	432.8	448.6
Mass change (%)	82.27	81.53	79.08	58.78	56.30	51.68
Residual mass (%)	9.55 (656.4 °C)	12.07 (653.5 °C)	15.96 (655.8 °C)	37.90 (643 °C)	40.17 (649.8 °C)	46.85 (675.0 °C)

*T*_onset_—initial temperature of the degradation curve; *T*_mid_—temperature with the middle of degradation curve; *T*_max_—temperature of highest degradation; *T*_end_—final degradation temperature.

**Table 3:** Decomposition temperatures at different levels of TG weight loss.

Sample Architecture	Resin Blend (ELO:GA:MLO)	Temperatures at Different Weight Loss (°C)				
		5 wt.%	10 wt.%	25 wt.%	50 wt.%	75 wt.%
FFFF	50:50:0	247.5	268.5	314.0	363.8	427.8
	50:40:10	247.5	272.5	299.4	368.1	443.7
	50:30:20	250.0	277.5	333.4	374.8	427.8
FBBF	50:40:10	275.0	297.5	359.7	423.3	-
BFFB		271.5	292.5	357.2	424.4	-
BBBB		302.5	362.5	375.0	400	-

## Data Availability

The data presented in this study are available on request from the corresponding author.
